# Porcelain gallbladder secondary to chronic cholelithiasis with filled gallstones: A case report and a minimal literature review

**DOI:** 10.1097/MD.0000000000045951

**Published:** 2025-11-07

**Authors:** Xiao-Fei Pan, Bilali Aizezi, Zhong Jia

**Affiliations:** a Department of General Surgery, The Third People’s Hospital of Yuhang District, Hangzhou, China; b Department of Thoracic Surgery, Xiang’an Hospital of Xiamen University, School of Medicine, Xiamen University, Xiamen, Fujian Province, China; c Department of Hepatobiliary Surgery, Affiliated Hangzhou First People’s Hospital, School of Medicine, West Lake University, Hangzhou, China.

**Keywords:** cholecystectomy, cholelithiasis, diagnosis, gallstone, porcelain gallbladder, surgery indication

## Abstract

**Rationale:**

Porcelain gallbladder (PG) is an academic term to define a special as well as rare condition of some gallbladder diseases. So far, some issues still remain a discussion, such as etiopathogenesis, clinical features, correlation with gallbladder cancer, and surgical indication. We described a typical PG case, and reviewed some English literature as well as relevant cases from our institutions, aiming to further illustrate some issues mentioned above.

**Patient concerns:**

Herein, we describe a 54-year-old obesity woman with hypertension and prediabetes who presented with recurrent abdominal pain and indigestion for 35 years, with diagnosis of chronic cholelithiasis.

**Diagnoses:**

She insisted on nonsurgical management for many years until a ring, high-dense shadow classically associated with PG, was found on computed tomography scan in the latest visit of the Emergency Department.

**Interventions:**

Therefore, she underwent a laparoscopic cholecystectomy.

**Outcomes:**

The patient recovered uneventfluly after surgery, and the post oprative pathology confirmed a PG without any cancer cells under microscopy. After a month of follow-up, she was very satisfied with the clinical outcomes, with no surgery-related complications.

**Lessons:**

PG has a potential though low risk of gallbladder cancerization. If patients’ condition permit, minimally invasive cholecystectomy is still a prioritized choice due to its safety and reliability as well.

## 1. Case report

**Figure 1.**
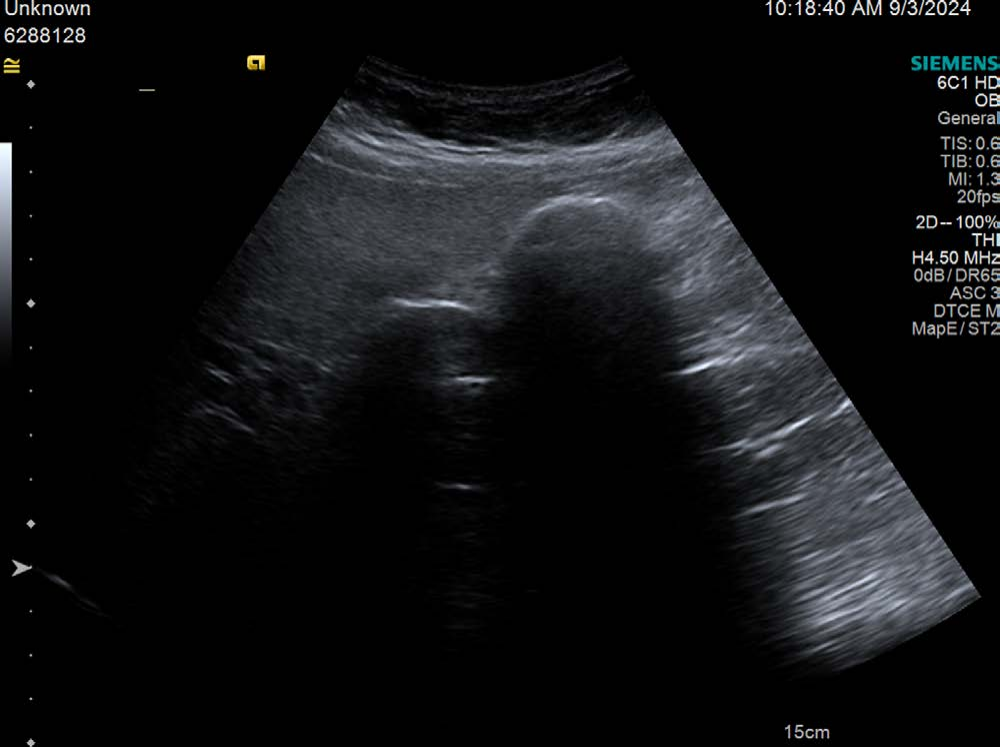
Apart from full-filled gallstones, abdominal ultrasonography also showed an obscure gallbladder wall with meniscus-shaped strong echo.

**Figure 2.**
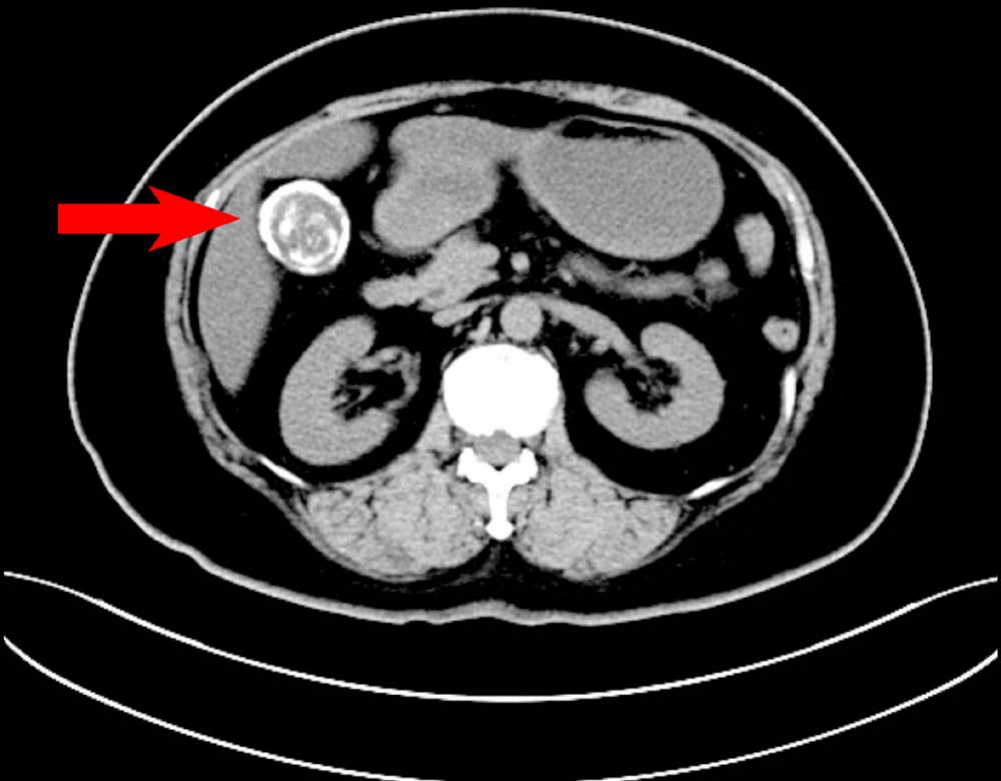
The latest general CT of the abdomen revealed an eggshell-shaped, evenly thickened cholecystic wall witha ring, high-dense shadow, suggesting diffuse calcification of the whole gallbladder wall classically associated with porcelain gallbladder. CT = computed tomography.

**Figure 3.**
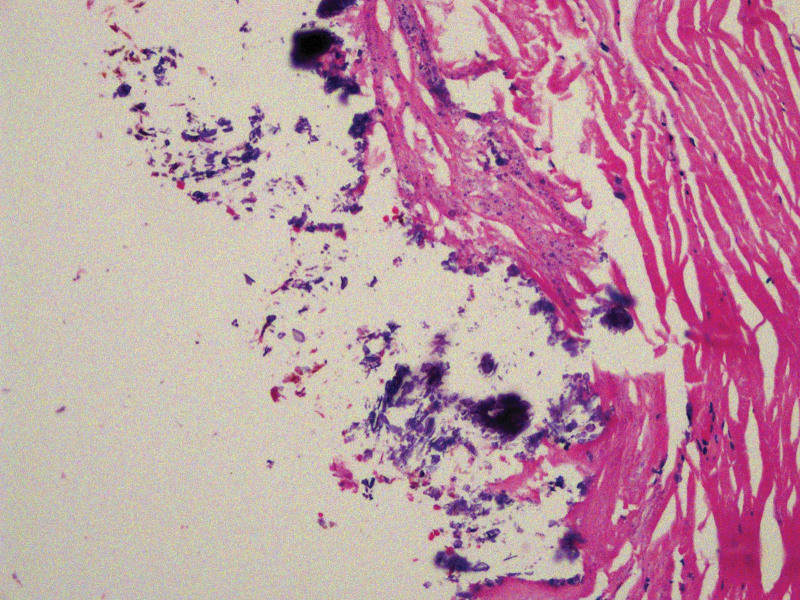
HE staining 10 × 20. Besides full-filled cholesterol stones, postoperative pathology also demonstrated a fibrotic gallbladder wall with atrophic mucosa and widespread transmucosal calcium deposition, which was in lining with characteristic features of porcelain gallbladder on pathology. HE = hematoxylin and eosin stain.

A 54-year-old obesity (hight: 158 cm, weight: 72.4 kg, body mass index: 29) woman with hypertension and prediabetes had been suffering from recurrent right upper abdominal pain and indigestion for 35 years before it struck again 1 day. Due to the similar symptoms described above, she had experienced lots of conservative treatments before, including Chinese Traditional Medicine, adimistraion of antibiotics and oral-taking painkiller, with diagnosis of cholelithiasis. Over time, the frequency of abdominal pain attacks was gradually reduced, but the therapeutic efficacy of indigestion was getting worse and worse. She denied any family history. Nothing was particular except for positive Murphy’s sign under physical examination. Laboratory testing showed a mildly elevated neutrophil percentage (75.2%, normal reference range: 50–70%) and a high blood glucose (6.8 mmol/L, normal reference range: 3.9–6.1 mmol/L). Apart from full-filled gallstones, ultrasonography also revealed an obscure cholecystic wall with meniscus-shaped strong echo (Fig. 1). Emergency general computed tomography (CT) of the abdomen revealed an evenly thickened gallbladder wall with ring, high-dense shadow, suggesting a diffuse calcification of the gallbladder wall classically with porcelain gallbladder (PG; Fig. 2, red arrow). The surgen-treating strongly advised the patient undergo a laparoscopic cholecystectomy, because the PG might have a risk of carcinoma transformation. The patient therefore underwent a laparoscopic cholecystectomy (operative time: 80 min, blood loss: about 10 mL), and was successfully discharged a day later. Besides cholesterol stones, pathology demonstrated a fibrotic cholecystic wall with atrophic mucosa and widespread transmucosal calcium deposition, without microscopic cancer cells (Fig. 3). After a month of follow-up, she was very satisfied with the clinical outcomes, with no surgery-related complications. The patient had given her written informed consent to publish her clinical case history and imaging data in public.

## 2. Discussion

Gallbladder diseases is very common in clinical practice, accounting for 1% to 10% of general population.^[1]^ PG an academic term to define a special form secondary to some gallbladder diseases, accounting for about 1% within surgically resected gallbladders. PG was well known as a high-risk factor gallbladder cancerization in the past, accounting for 7% to 60%.^[2]^ Nowadays, more and more evidences provide a subversion of the prior viewpoint, with only 0.8% to 6% incidence of gallbladder cancerization. Thus, the completely different results become a hot topic arousing people’ great interesting.

PG is a visual description for gallbladder calcification and/or transmucosal calcium deposition, presenting a hard, brittle texture and pale blue appearance similar to porcelain. Over 95% of PG is co-existed with chronic cholelithiasis, especially with full-filled gallstones. For this reason, it is generally believed that PG is caused by long-lasting irritation of chronic inflammation. Additionally, other multiple approaches might also involve its development, including cholecystic wall ischemia due to compression of gallstones, and calcium expansion associated with diffusion phenomenon.^[3]^ Characterized CT findings, such as eggshell-like high-dense shadow, play an important role in its diagnosis and differential diagnosis.^[4]^ Of note, gallbladder calcification is also seen in other entities, including gallbladder cancer, submucosal stones, and stones at the mucosa. Therefore, the final conclusion depends on its pathology.

As understanding of PG deepens and laparoscopic technology continues to advance, the approach to PG surgery has evolved accordingly. In the past, it was generally believed that the risk of cancer transformation in PG was as high as 22%,^[5]^ with malignancy being a result of the gradual accumulation of chronic inflammation.^[6]^ Before the development of laparoscopic technology, it was generally believed that all cases of PG should undergo open cholecystectomy, or even extended resection. However, as laparoscopic techniques gradually advanced in the field of surgery, it became recognized as a reliable minimally invasive approach. Despite this, many scholars still considered PG a relative contraindication for laparoscopic surgery.^[7]^ Whether or not to perform laparoscopic cholecystectomy largely depends on the surgeon-treating’s expertise with laparoscopic techniques and the communication between the doctor and the patient. Over time, retrospective studies on large PG cohorts have shown that the risk of gallbladder cancer in patients with gallbladder wall calcifications is lower than previously anticipated.^[7]^ As laparoscopic technology widely become accepted, an increasing number of patients are undergoing laparoscopic cholecystectomy for PG. In fact, with the growing acceptance of laparoscopic techniques, this approach is considered a safe and reliable minimally invasive option and should be the first choice for treatment. The key challenge lies in determining before surgery whether the condition is simple PG or if cancer transformation or suspected malignancy has occurred. Literature suggests that identifying cancer transformation in PG primarily relies on a clear understanding of the patient’s medical history, tumor markers, and imaging features.^[2]^ For instance, CT scans showing abnormal tumor signals such as irregular thickening of the gallbladder wall, uneven enhancement, or elevated serum tumor markers can be indicative of malignancy. For patients without evidence of cancer transformation, laparoscopic cholecystectomy is the preferred approach. Conversely, in cases with suspected cancer transformation, open extended resection should be considered. Regardless of the surgical approach, routine intraoperative frozen section examination should be performed to confirm the presence or absence of malignancy. If PG malignancy is unexpectedly discovered during surgery, the surgical approach should be adjusted accordingly. For patients with PG malignancy discovered postoperatively, further surgery should be considered based on the tumor staging of PG.

Some studies reported 5% to 22% non-gallbladder cancer specimens were found with PG, suggesting a possible correlation between them. Nevertheless, in our opinion, PG is just a potential though low risk of gallbladder carcinoma transformation rather than a pre-carcinoma lesion. Thus, PG known as an absolute indication for surgery should have a little change in this point. For example, for PG patients at high risk of surgery, surgery had better to be avoided, and be instead with watch-and-wait. Eight; whatsoever, making an individualized plan for treatment through weighing the pros and cons of each therapeutic option and appropriate assessment of patients’ general condition is the key to the clinical success.^[8]^

All in all, PG could lead to contraction dysfunction of the gallbladder, bile metabolism disorder and potential risk of gallbladder cancer. In the past, surgery was the preferred treatment for PG, but it is currently considered that the cancer risk is relatively low. Therefore, for elderly patients with high surgical risks, a wait-to-see approach may be considered. However, for patients with comorbidities such as diabetes, hypertension, those over 50, obesity, refrequent pain attacks, or suspected malignancy, active cholecystectomy should still be recommended. For PG without evidence of malignancy, laparoscopic cholecystectomy remains the first choice due to its minimally invasive trauma, safety, and effectiveness.

## Author contributions

**Conceptualization:** Bilali Aizezi.

**Data curation:** Bilali Aizezi.

**Investigation:** Xiao-Fei Pan.

**Resources:** Xiao-Fei Pan.

**Supervision:** Zhong Jia.

**Writing – original draft:** Xiao-Fei Pan.

**Writing – review & editing:** Zhong Jia.
